# Nutritional support for pressure injury: a bibliometric analysis of research trends

**DOI:** 10.3389/fnut.2026.1839197

**Published:** 2026-07-02

**Authors:** Na Liu, Rui Liang, Jia Zhu, Erge Jia, Wenwen Tong, Xueyan Li, Weiying Zhang, Runv Zhou

**Affiliations:** 1 Department of Emergency, Shanghai East Hospital, Shanghai, China; 2 School of Medicine, Tongji University, Shanghai, China; 3 Surgical Intensive Care Unit, Shanghai East Hospital, School of Medicine, Tongji University, Shanghai, China; 4 Department of Nursing, Shanghai East Hospital, School of Medicine, Tongji University, Shanghai, China; 5 Department of Nursing, Shanghai East Hospital, Shanghai, China

**Keywords:** bibliometrics, nutrition, nutritional support, pressure injury, trend analysis

## Abstract

**Introduction:**

Pressure injury (PI) is a common complication closely associated with malnutrition. Nutritional support is critical for PI prevention and healing, but the overall research trends in this field remain unclear.

**Purpose:**

This bibliometric analysis aimed to explore trends in research on nutritional support for Pressure Injury (PI) patients, providing insights for healthcare professionals and researchers.

**Methods:**

This study is a bibliometric analysis. Relevant English articles were retrieved from the Web of Science Core Collection (WOSCC) and PubMed databases, with the search period spanning from the establishment of each database to February 2026. CiteSpace was employed to analyze the articles.

**Results:**

A total of 1,380 publications were included in this study. The number of published articles showed a consistent growth trend, with a significant increase during 2019–2020. Regarding research output, the United States remains the core contributing country in this field, and Griffith University (Australia) ranks first in institutional output; Clinical Nutrition is the journal with the most published relevant studies, and Thomas DR is the most prolific author. Keyword clustering analysis identified 10 core research themes, including Pressure Injury, enteral nutrition, traumatic brain injuries, nursing home, retrospective study, risk factors, geriatric nutritional risk index, nutritional status, and intensive care unit. Emerging research hotspots are: palliative care, wound healing, and nutritional risk stratification factors in critically ill patients. The current research focus has been significantly concentrated on acute or critically ill patient populations.

**Conclusion:**

Nutritional support in Pressure Injury (PI) management has garnered growing research attention. Future studies should emphasize multidisciplinary collaboration to develop personalized, evidence-based protocols tailored to diverse patient conditions. Translational research is essential to optimize outcomes, especially for high-risk populations, and targeted nutritional strategies remain pivotal for effective PI management.

## 1 Introduction

Pressure Injury (PI) is localized skin and/or underlying tissue injury, usually over bony prominences, caused by pressure alone or combined with shear, with several contributing factors whose significance remains unclear (1). Latest epidemiological data shows the global prevalence of PI is 12.8% (2), and the hospital-acquired PI incidence is 8.5% among hospitalized adults (3). PI exerts deleterious effects on patients, including pain (4), infections, increased healthcare expenditures (5), and even result in death (6, 7). Nutritional factors are independent risk factors for Pressure Injuries (1). The 2025 clinical practice guideline jointly published by NPUAP, EPUAP, and PPPIA recommends nutritional support for PI management (1) as nutrition plays a significant role in PI prevention and treatment by promoting skin integrity and supporting tissue repair in vulnerable populations (3, 8).

Despite numerous studies on nutritional support for PI treatment, traditional research methods (literature reviews, meta-analyses, and data mining) may introduce bias and miss key information, failing to accurately reflect the field's comprehensive landscape (9, 10). Additionally, existing randomized controlled trials (RCTs) have methodological flaws: improper timing of nutrient interventions (11), inadequate randomization (12), small sample sizes (4), and a lack of long-term cohort studies (11). Thus, evidence for nutritional support in PI management remains insufficient, with unclarified research themes and trends hampering clinical practice and targeted research.

In this study, the co-occurrence analysis was performed using the Cite Space software. By visualizing and analyzing data such as authors, research institutes, keywords, countries, cited literature, and cited authors, the structural pattern of the research field is revealed (13). This methodological approach helps to draw scientific and accurate conclusions. Cite Space software utilizes dynamic mapping to present results, ultimately mapping abstract data in two-dimensional or three-dimensional graphs. It provides knowledge mapping from a multidimensional, time-series perspective (14). The software can reveal the evolution and dynamics of a research field, analyze its current status, and identify research hotspots (13). It can also conduct quantitative and qualitative analysis, explore the knowledge structure, and detect potential new trends in the field. Finally, it can provide support and references for in-depth, relevant research (15).

## 2 Data and methods

### 2.1 Study design

This study is a bibliometric analysis. It aims to quantitatively evaluate publication trends, research hotspots, and knowledge evolution of nutritional support for pressure injury. The flowchart only illustrates the literature screening process for bibliometric analysis.

### 2.2 Literature search

A comprehensive literature search was performed in Web of Science Core Collection (WOSCC) and PubMed from database inception to February 28, 2026. WOSCC provides complete citation data, standardized metadata, and high-quality indexed journals that are widely recognized as suitable for bibliometric visualization and trend analysis. PubMed comprehensively covers clinical nutrition, wound care, and critical care literature, ensuring sufficient inclusion of clinically relevant studies. The combination of WOSCC and PubMed guarantees the authoritativeness, completeness, and reproducibility of the data source for this bibliometric analysis. Only English articles were included. The complete, reproducible search strings for both databases, including the exact date of search (February 28, 2026), the database interface versions, and the validated Boolean syntax with appropriate field tags, are provided in Supplementary File 1. The key search parameters are summarized in Table 1. Readers are strongly advised to consult Supplementary File 1 for the exact executable syntax.

**Table 1:** Literature search strategy.

Search ID	Search query	Results (WOS)	Results (PubMed)
#1	See Supplementary File 1	436,713	1,353,531
#2	See Supplementary File 1	16,754	9,229
#3	#1 AND #2	953	698
#4	#3 AND PUBLICATION DATE RANGE−2026 AND LA = (ENGLISH) AND DT = (PAPER OR REVIEW OR RCT)	864	646

### 2.3 Inclusion and exclusion criteria

Original articles and review articles relevant to PI and nutritional support were retrieved. The following publications were excluded: conference papers or abstracts, unpublished articles, duplicate publications, articles not related to the topic, pathological reports, and qualitative studies.

### 2.4 Data export, cleaning, and deduplication

**Figure 1:**
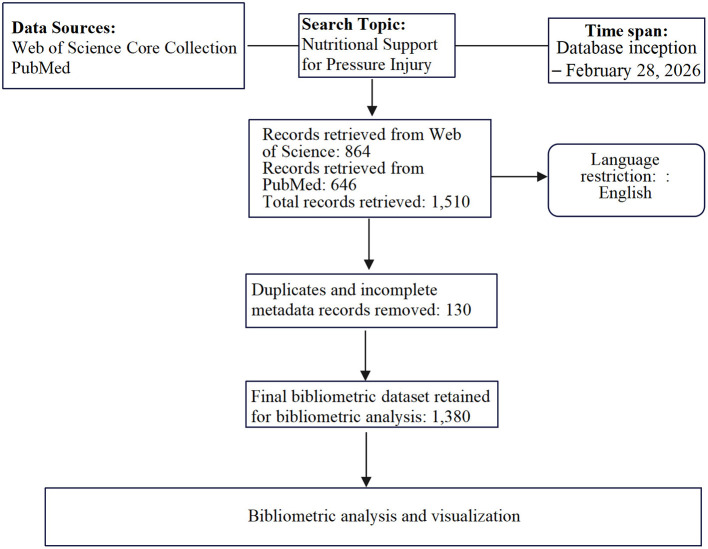
Data retrieval and selection workflow for bibliometric dataset construction, which includes records retrieved from WOSCC and PubMed.

Publications matching the search strategy were be selected and exported in Plain Text format, with the full text and the referenced bibliography recorded. It is imperative to verify that the exported.txt file contains all designated fields. For instance, the CR field must include the bibliography list. Subsequently, data cleaning was performed to check the integrity and accuracy of all key fields, including titles, authors, years, keywords, and references. Records with missing core information, unrecognizable characters, or incomplete reference data were excluded. Duplicate records retrieved from WOSCC and PubMed were removed using database tools and manual cross-checking to eliminate redundant data and ensure the reliability of subsequent analysis. Records from WOSCC and PubMed were merged *via* reference management software. Duplicates were removed by matching DOI, title, and authors, prioritizing DOI. Two reviewers independently screened titles/abstracts; disagreements were resolved by consensus. The detailed screening and selection process for publications is presented in Figure 1. After applying language (English only) and publication-type (original/review articles only) restrictions, 953 WOSCC records were reduced to 864, and 698 PubMed records were reduced to 646, excluding non-English, non-research, and non-eligible publication types, and the final dataset of 1,380 publications included for bibliometric analysis. All data were traceable and internally consistent.

### 2.5 Bibliometric analysis

This study followed standardized bibliometric procedures and validated CiteSpace methods to ensure analytical rigor and reproducibility (13, 14). Bibliometrics is a field of study that utilizes quantitative and statistical methods to analyze scientific literature to reveal research trends. In this study, the Cite Space 6.1.R1 software (15) was utilized to visualize and analyze research on nutritional support for PI from the establishment of each database to February 2026 in the Web of Science Core Collection and PubMed databases. CiteSpace parameters were set as follows: time slicing = 1 year; node type = authors, institutions, and keyword; The cosine algorithm was applied for network clipping.G-index (k = 100) was selected to extract high-impact and high-centrality nodes-this k-value balances the extraction of influential research nodes and the integrity of bibliometric data, avoiding omission of key information while preventing network redundancy; pruning sliced networks and pruning merged networks were used to simplify the network structure while retaining core information. These settings ensured the stability and reliability of the clustering results. We constructed collaborative networks, identified keywords with citation bursts, and analyzed changes in research hotspots and frontiers in this field. In this study, the Nvivo12 software was utilized to analyze the keywords present in the exported literature and generate a word cloud. The size of a word in the word cloud is indicative of its frequency of occurrence. This process serves to verify the analysis of keywords by Cite space software. The parameters of the Nvivo software are as follows: the material range is the plain text document of the exported literature; the minimum word length is six letters; and the display is set to the first 100–200 high-frequency words. The process of literature screening and analysis is illustrated in Figure 1.

## 3 Results

### 3.1 Publication trends

**Figure 2:**
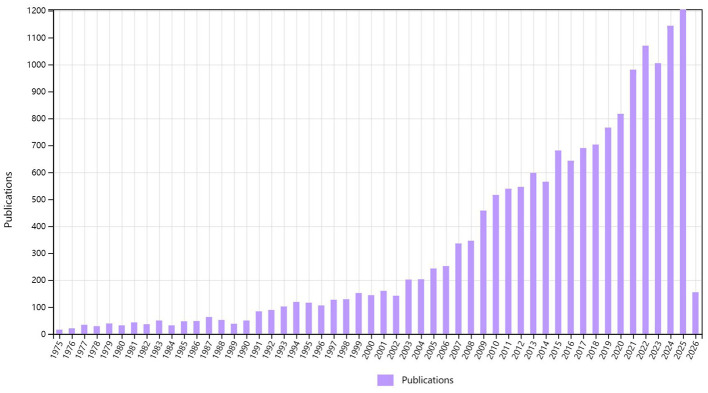
Histogram of annual number of articles issued in the field of pressure injury (PI) research.

**Figure 3:**
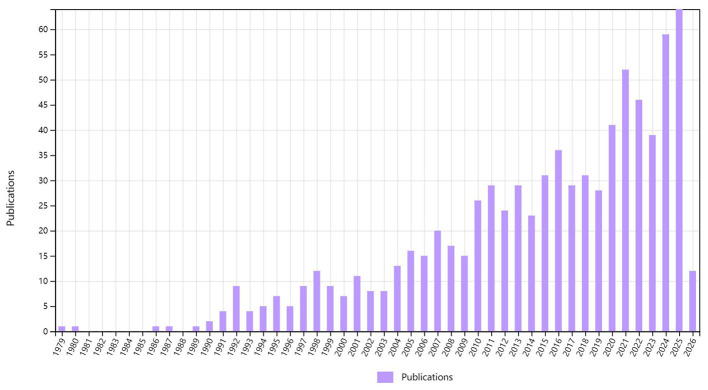
Histogram of annual publications in the field of PI nutrition support.

**Figure 4:**
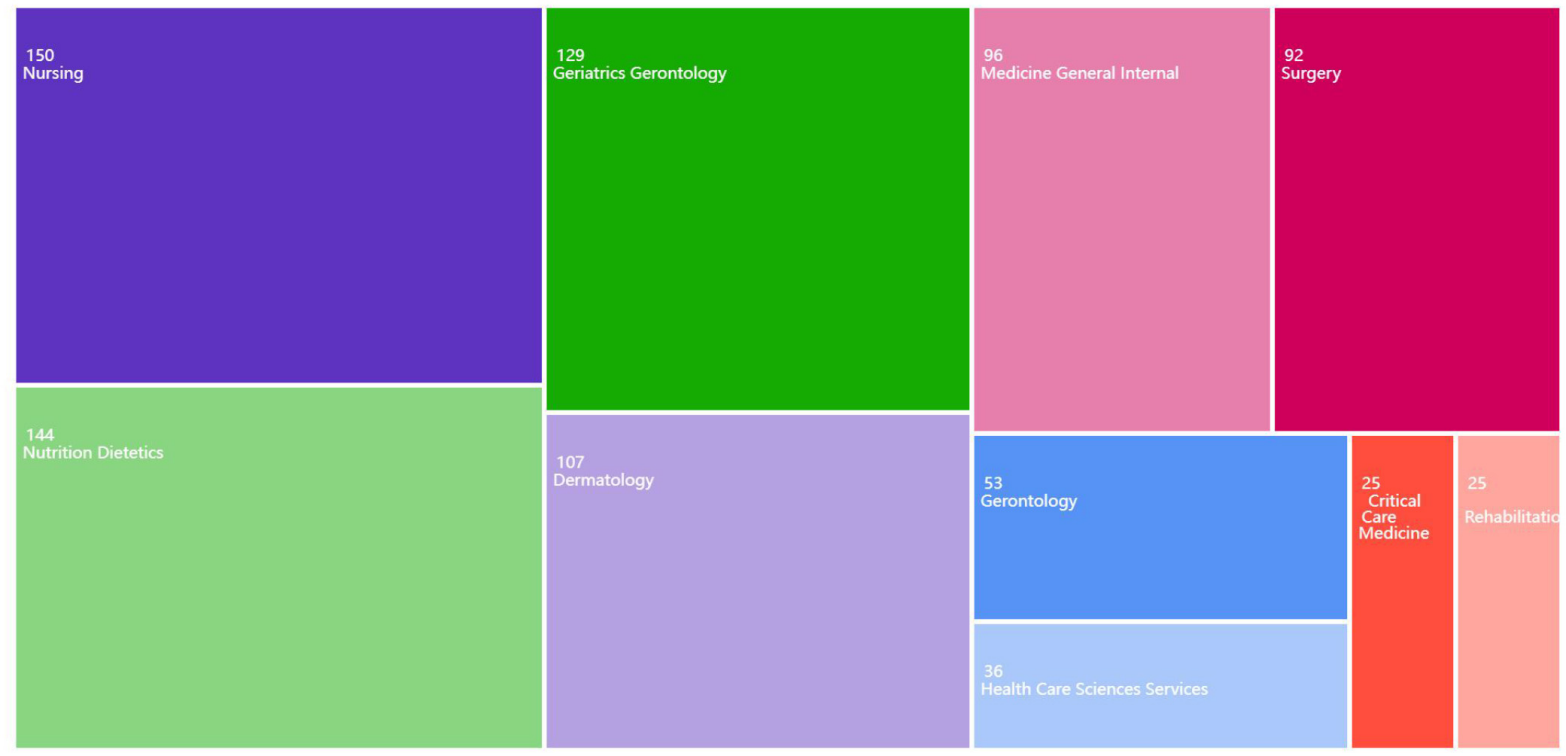
Distribution of research areas covered in PI nutritional support studies.

Up to February 2026, publications on Pressure Injury (PI) and nutritional support have increased steadily in the Web of Science Core Collection and PubMed databases, with the number peaking in 2025. Publication trends are detailed in Figures 2, 3 and active research areas are depicted in Figure 4. Among these areas, Nursing ranked first, followed by Nutrition and Geriatrics. The number of publications across different research areas is shown in Figure 4.

### 3.2 Nations and institutions with the highest number of publications

**Figure 5:**
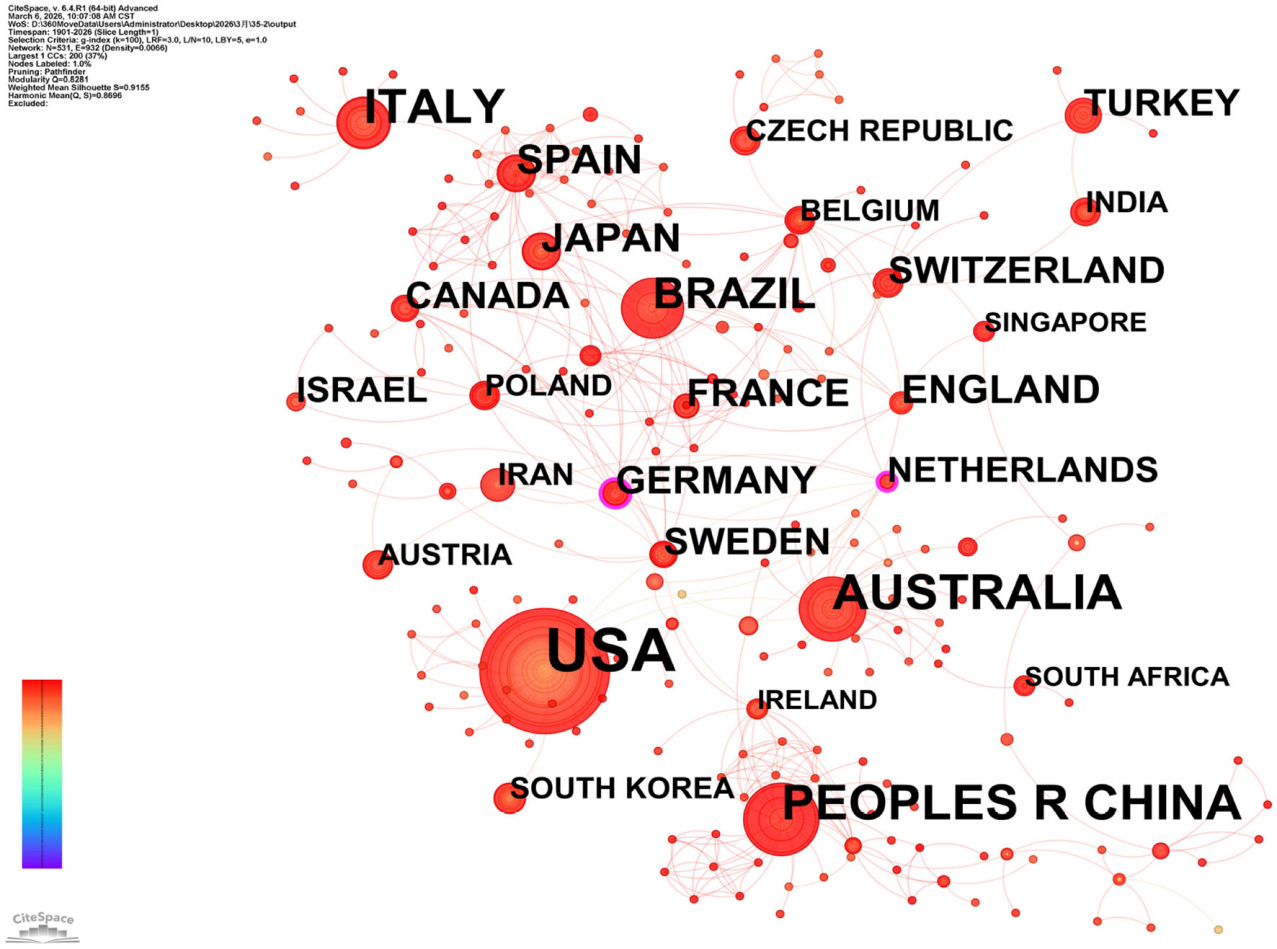
Country cooperation network in the area of PI nutritional support.

**Figure 6:**
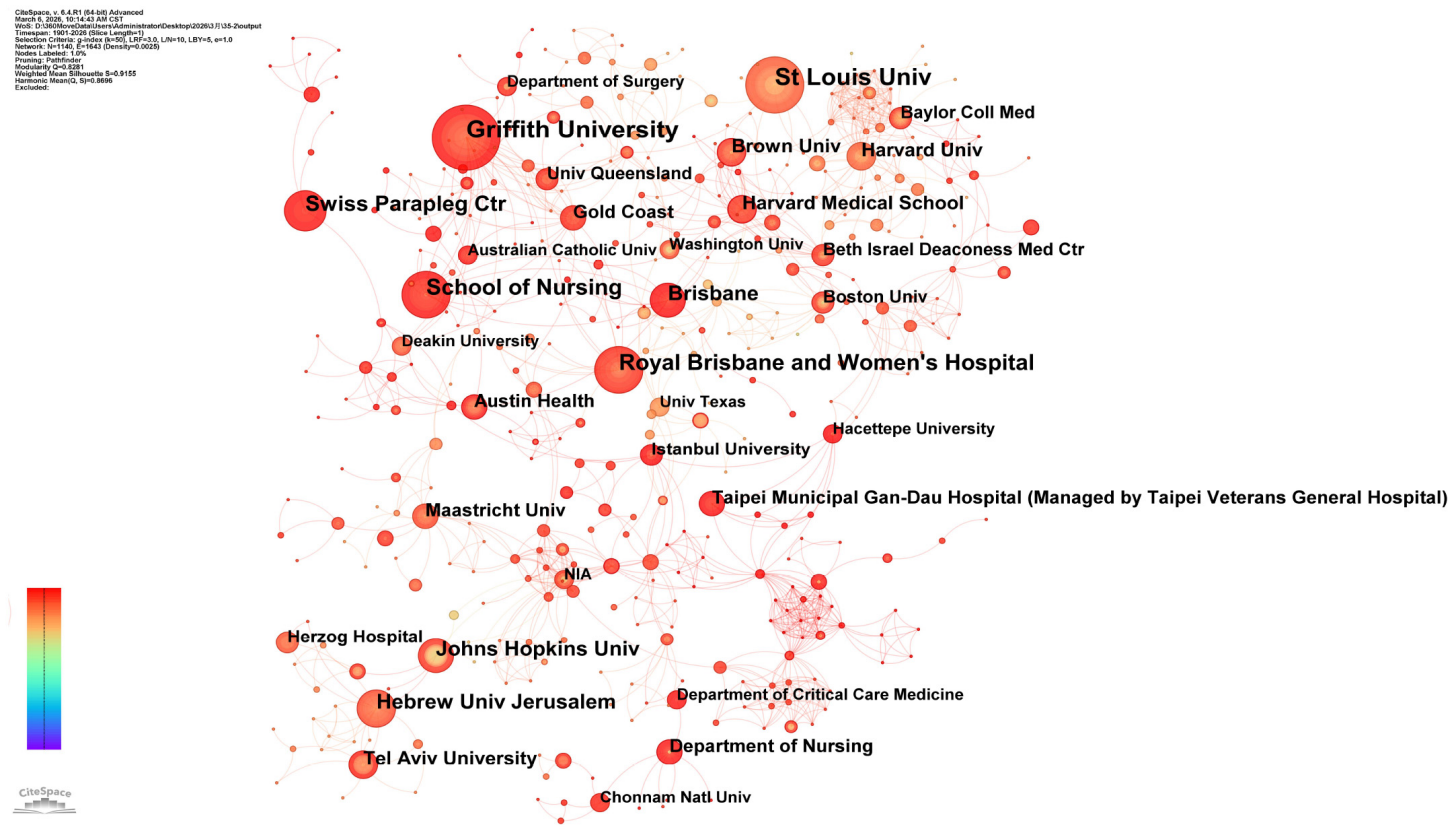
Institutional cooperation network in the area of pressure injury (PI) nutritional support.

The United States ranked first in publication volume (*n* = 381), followed by Australia (*n* = 106) and Italy (*n* = 91); Germany and the Netherlands play a pivotal role in the collaborative network (centrality >0.1). The top three institutions were Griffith University (*n* = 21), St Louis University (*n* = 18) and Royal Brisbane Women's Hospital (*n* = 15). As illustrated in the collaboration networks, institutions from the United States, Europe, and Australia maintain the most frequent collaborative ties. Taipei Municipal Gan-Dau Hospital and Chonnam National University have significantly enhanced their academic influence through international collaborations with Harvard University. Institutional collaborations in this field are highly active and exhibit distinct geographic clustering, with cross-continental collaborations primarily occurring among top-tier academic institutions. The national collaboration network is visualized in Figure 5. The institutional collaboration network shown in Figure 6.

### 3.3 Academic journals and contributing authors

**Figure 7:**
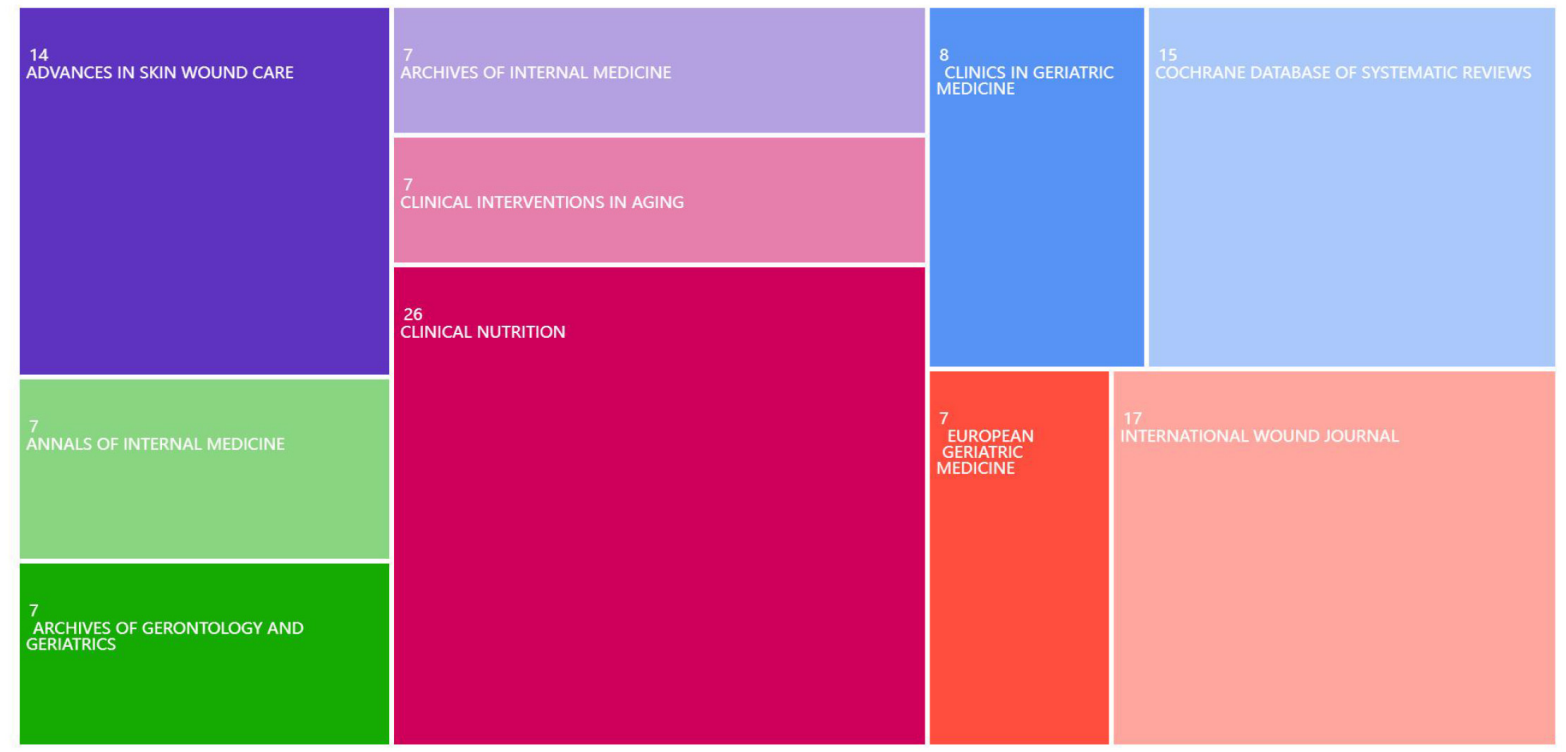
Top 10 journals with publications in the field of PI nutrition support.

**Figure 8:**
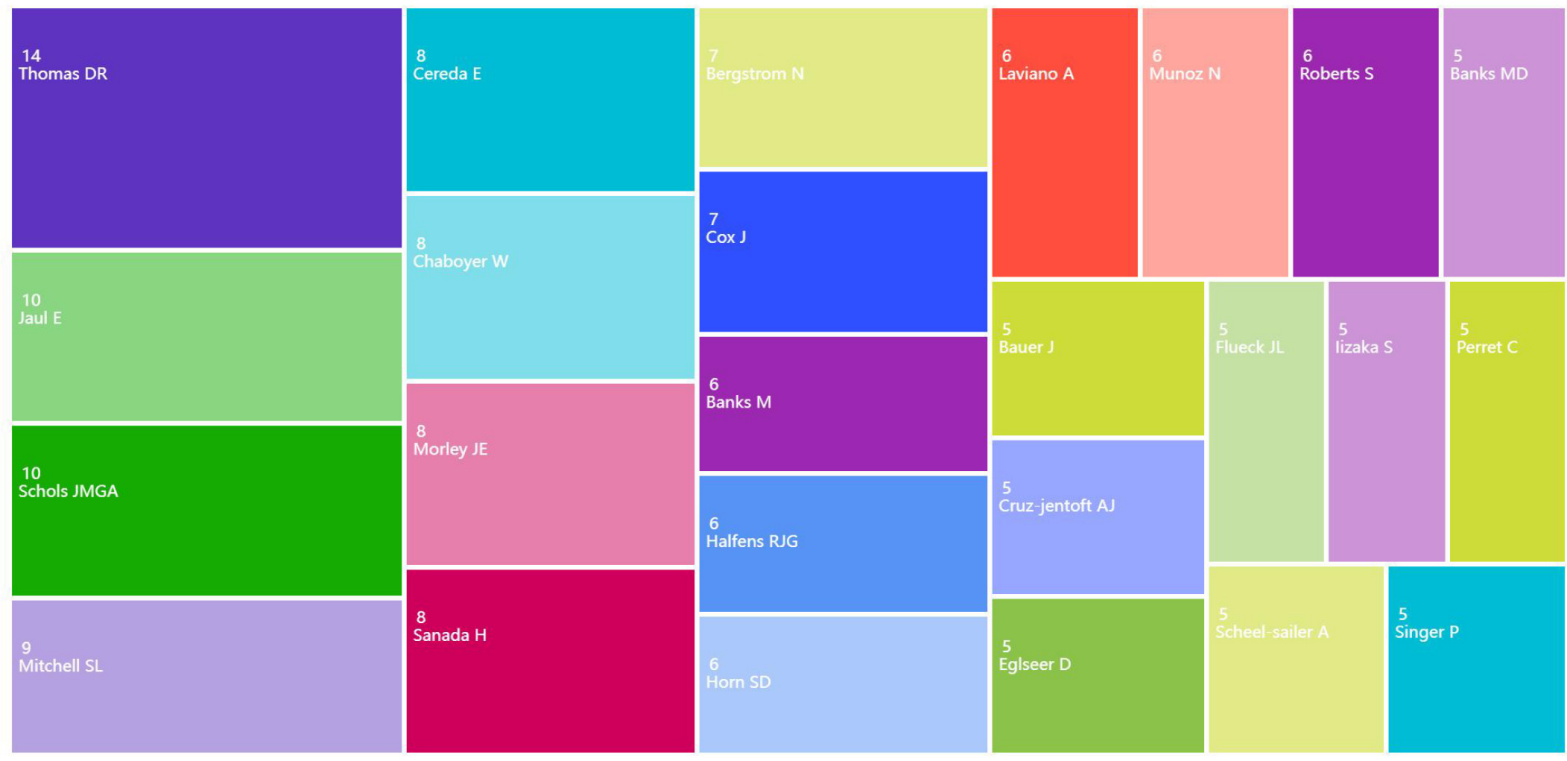
Top 10 authors with publications in the field of PI nutrition support.

**Figure 9:**
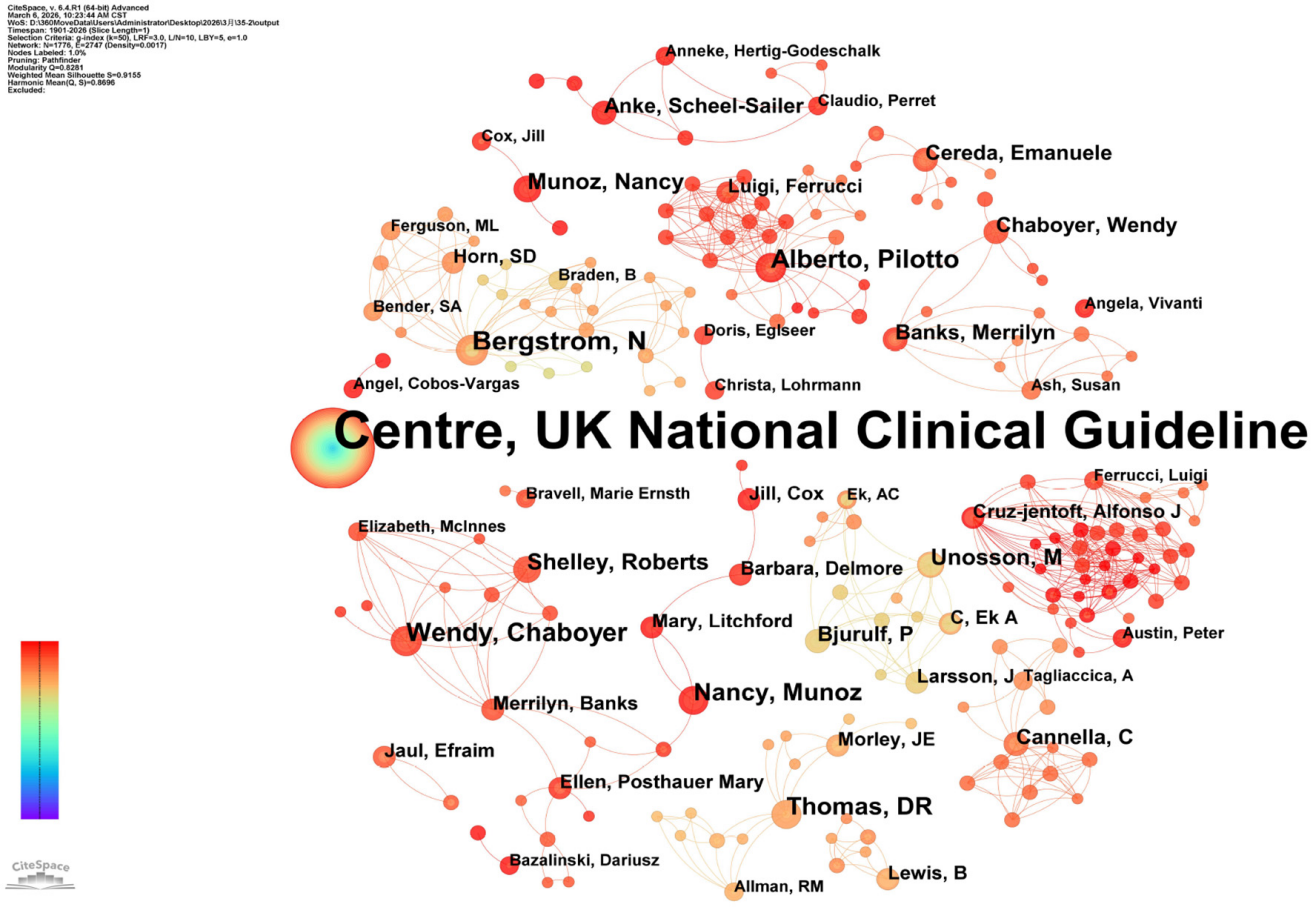
Author collaboration network for PI nutrition support.

Up to February 2026, the top three journals with publications on PI nutritional support were the Journal of *CLINICAL NUTRITION* (26), *International Wound Journal* (17), and *COCHRANE DATABASE OF SYSTEMATIC REVIEWS* (15). Among the most frequently cited publications, the ESPEN guidelines (16) establish nutritional support as the central treatment for patients with multimorbidity. The most prolific author was Thomas, David R. The top three co-cited authors were all from Europe. The research collaboration was dominated by the research team led by Cruz-Jentoft Alfonso J, followed by the research team led by Alberto, Pilotto. The journal, author and collaboration network, and journal citation data are displayed in Figures 7–9.

### 3.4 Co-occurrence analysis of keywords

**Figure 10:**
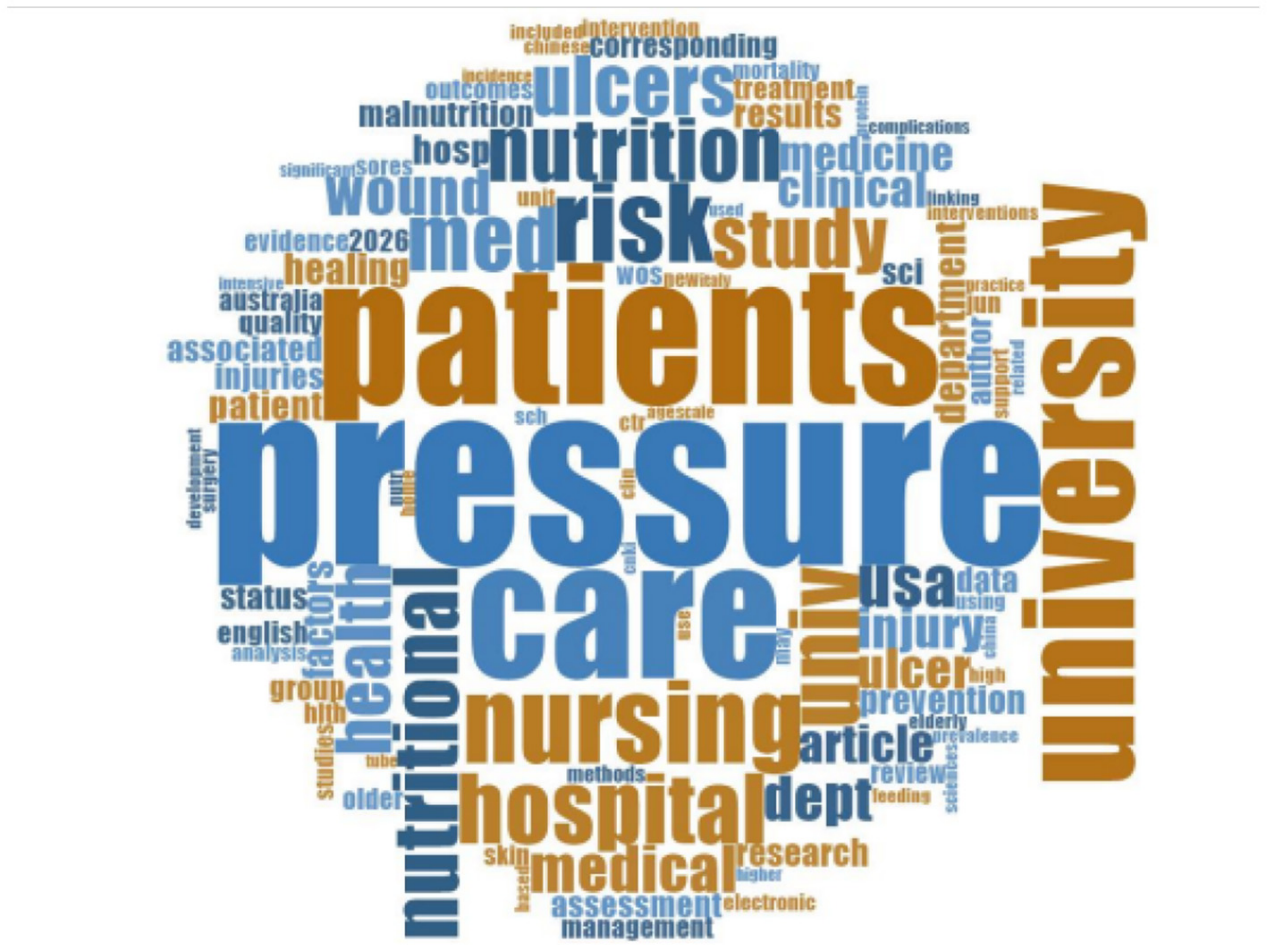
High-frequency keyword word cloud in the PI nutritional support domain.

**Figure 11:**
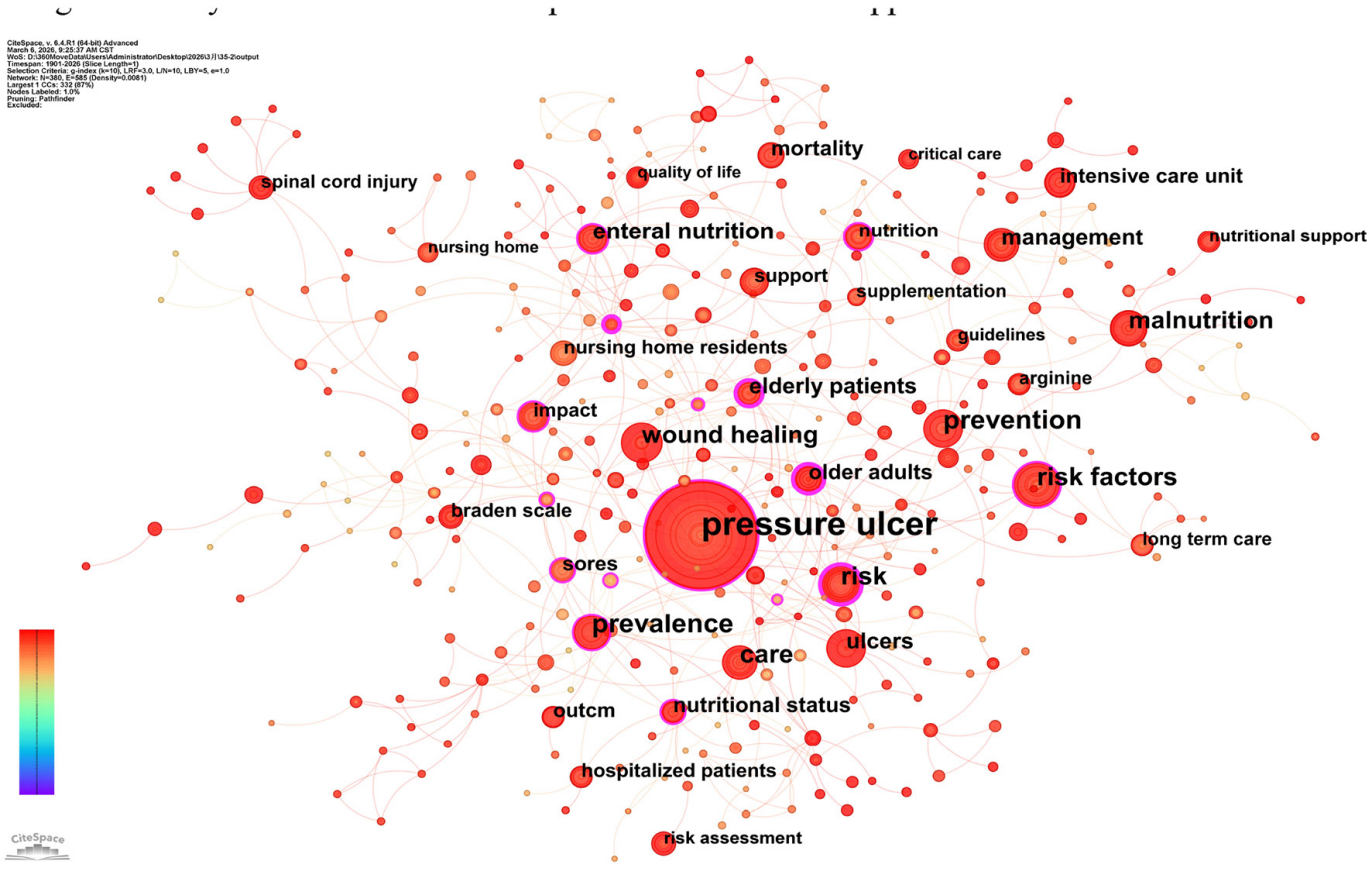
Keyword co-occurrence map for the PI nutrition support domain.

The co-occurrence analysis of keywords demonstrated that PI was the most frequent keyword. The top three keywords in terms of centrality were: elderly patients, risk factors, and advanced dementia. The keyword cloud provides an overview of the core themes of Pressure, patients, and the care. The co-occurrence of high-frequency words such as “Pressure Injury,” “risk factors,” “elderly patients,” and “enteral nutrition”. The keyword clouds and co-occurrences are presented in Figures 10 and 11.

### 3.5 Clustering analysis of keywords

The clustering analysis of keywords demonstrated that 10 clusters were generated based on the LLR algorithm, The overall keyword co-occurrence network had a Modularity *Q* of 0.8281 (*Q* > 0.3 indicates significant community structure), a mean silhouette value of 0.9155, and a network density of 0.0081 and all contour values were higher than 0.76, indicating a clear structure (17). The largest cluster was basic prevention and control of Pressure Injurys (contour value: 0.962, 29 keywords), with core focuses on risk assessment, prevention of hospital-acquired Pressure Injurys and care bundle strategies, which represents the most systematic research direction in this field. The cluster of micronutrients and wound healing (contour value: 0.986, 2003) focused on vitamin D, ascorbic acid (vitamin C), and trace elements in wound healing of patients with diabetic foot ulcers and hip fractures. The cluster of critical care and enteral nutrition (contour value: 0.756, 2016) focused on enteral/parenteral nutrition regimens, clinical practice guidelines for ICU patients and nutritional management of patients under prone ventilation. The cluster of palliative care and advanced-stage intervention (contour value: 0.957, 2018) covered prevention and control of medical device-related Pressure Injurys, arginine supplementation, and jejunal tube feeding support.

**Figure 12:**
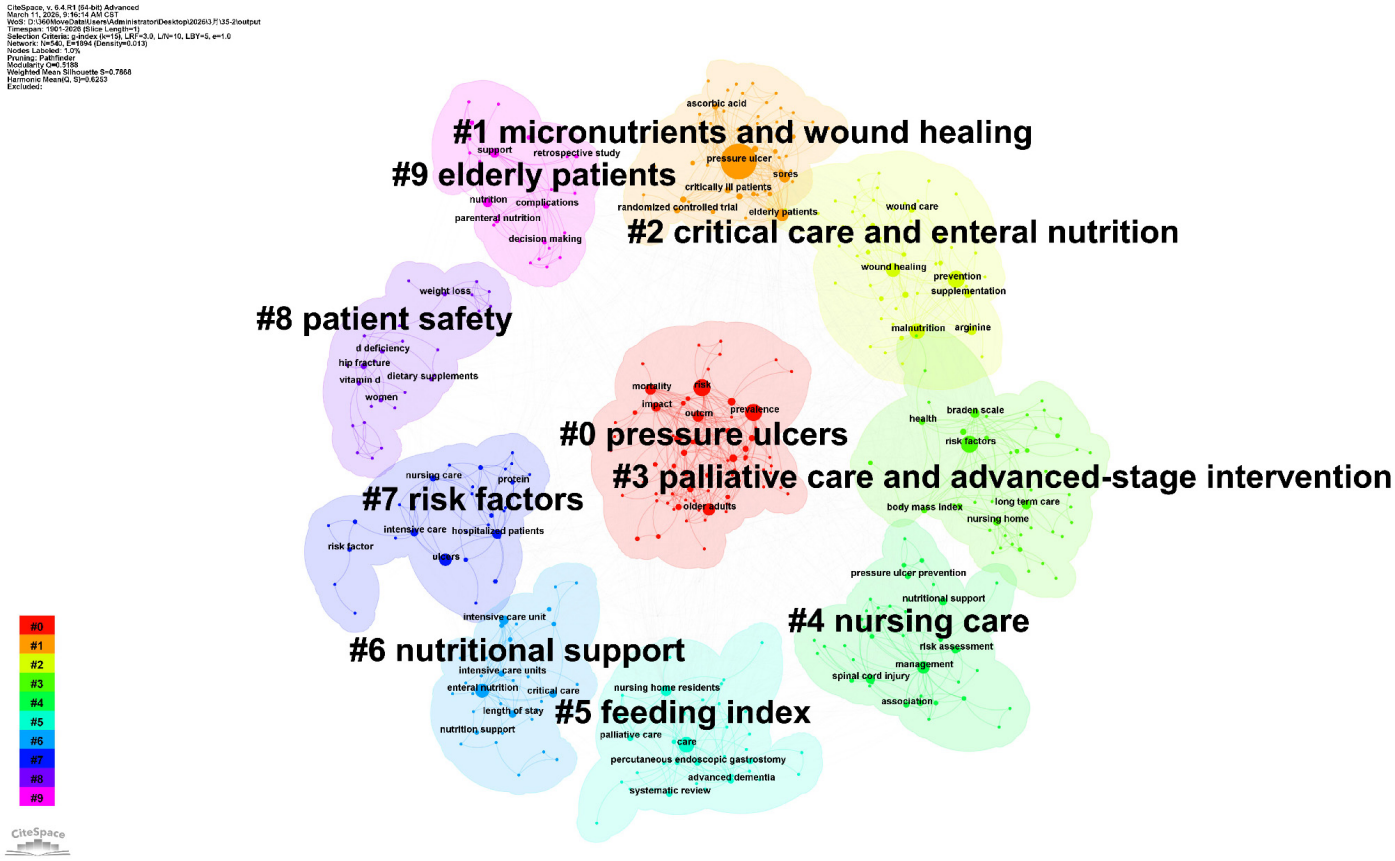
Clustering analysis of high-frequency keywords in the field of PI nutritional support.

Research evolution showed distinct phased characteristics: 1994–2010 focused on basic nutritional assessment and risk identification; 2011–2015 centered on nursing standardization and evidence translation; since 2016, research has expanded toward precision nutrition in critical care, palliative care and micronutrient metabolism monitoring. The results of keyword clustering analysis and cluster structure visualization are presented in Figure 12.

### 3.6 Research hotspots and frontiers (citation burst analysis)

CiteSpace was used to identify the research hotspots and frontiers of nutritional support for PI, and a timeline view of this research field was plotted—with the X-axis representing the literature publication year and the Y-axis indicating cluster numbers. A total of 25 frequently cited keywords were identified in the field of nutritional support for PI, and the dynamic frontier evolution graph reflects the shifts in research focus of PI nutritional support over the study period. Citation burst analysis identifies keywords with a sharp increase in citation frequency during specific periods. This metric reflects temporary surges in research attention rather than established future directions. With this caveat, the analysis showed that the key emergent keywords in recent years were intensive care (2021–2026); while the keywords with the latest and ongoing citation bursts (2022–2026) include ulcers, risk factor, and the 2024–2026 emerging burst keywords wound healing and palliative care are among the latest areas that have garnered increased attention.

## 4 Discussion

In recent years, a growing number of studies have focused on nutritional support for pressure injury, with systematic reviews and meta-analyses confirming the role of protein, micronutrients, and early nutritional intervention in PI prevention and wound healing. However, most existing studies are clinical trials or narrative reviews; few studies have provided a comprehensive, visualized, and time-sequenced analysis of the entire field.

### 4.1 Overall publication and collaborative characteristics

Research on nutritional support for PI has maintained a continuous growth trend in the international academic community, with the research focus gradually shifting from early disease risk assessment to individualized nutritional intervention, and a full-cycle management model centered on “risk identification-assessment-intervention” is initially taking shape. Multidisciplinary and interdisciplinary collaboration has become an important development trend in this field, and the exploration of personalized nutritional support strategies such as micronutrient intervention has gradually become the core focus of subsequent research.

Bibliometric analysis revealed a sustained growth in publications on nutritional support for Pressure Injury (PI) from database inception to February 2026, with a notable surge in 2019–2020 and a publication peak in 2025 (Figure 2). This growth pattern reflects the increasing global attention to PI-related nutritional research, which is closely related to the aging population structure, the rising incidence of chronic diseases, and the continuous improvement of clinical nutrition research systems.

This trend is driven by global aging, rising demand for standardized PI management, and boosted by critical care nutrition research during the COVID-19 pandemic^6^ and the 2025 NPUAP/EPUAP/PPPIA Guideline release (1). The United States, Australia and Italy ranked top in publication volume for their high research investment and early evidence-based practice (18), while Germany and the Netherlands (centrality >0.1, Figure 4) played a core role in international collaboration for their contributions to European geriatric nutrition research and ESPEN guideline formulation (1, 16).

Institutional collaboration networks exhibited obvious geographical aggregation characteristics (Figure 6), with Griffith University, St Louis University and Royal Brisbane Women's Hospital as the top three contributing institutions. The stable cooperative relationships formed between these institutions laid a solid foundation for the continuous output of high-quality research in this field. Griffith University laid a foundation for clinical translation *via* local collaborations (19), and transnational partnerships (e.g., Taipei Municipal Gan-Dau Hospital with Harvard University) have become a key driver for global academic influence and clinical experience popularization.

### 4.2 Core research entities: journals and authors

Up to February 2026, the *Journal of Clinical Nutrition, International Wound Journal* and *Cochrane Database of Systematic Reviews* were the top three publishing journals (Figure 7), representing core academic platforms in clinical nutrition and wound management; the high volume in the Cochrane Database highlights the critical role of high-level evidence in guiding research and practice (4). Thomas DR was the most prolific author, and European scholars dominated the co-cited author list, shaping the field's research paradigm. The collaboration network was led by Cruz-Jentoft Alfonso J and Alberto Pilotto's teams (Figure 9), whose geriatric nutritional risk research supported PI nutritional support standardization (16, 20); Chaboyer Wendy made innovative contributions to critically ill patient intervention and health economic evaluation, with her work offering reference for healthcare resource optimization (19, 21).

The distribution of core journals reflects the disciplinary attributes and research orientation of this field: clinical nutrition journals focus on the nutritional mechanism and intervention effect of PI, wound care journals pay attention to the clinical application and management strategy of nutritional support, and evidence-based medicine journals provide high-level research evidence for the formulation of clinical guidelines. Thomas DR was identified as the most productive author in this field, and the research teams led by European scholars such as Cruz-Jentoft Alfonso J and Alberto Pilotto occupied the core position in the co-citation network, indicating that European research groups have long played a leading role in promoting the development of PI nutritional support research.

### 4.3 Research hotspots based on keyword analysis

#### 4.3.1 Keyword co-occurrence characteristics

PI was the most frequent keyword, with elderly patients, risk factors and advanced dementia ranking top three in centrality (Figures 7 and 8), clarifying the core high-risk research population—these groups have high malnutrition rates and poor wound healing prognosis (16, 22). The co-occurrence of Pressure Injury, risk factors, elderly patients and enteral nutrition suggests targeted nutritional support for high-risk populations as the core direction, indicated by the 2025 NPUAP/EPUAP/PPPIA Guideline^1^.

Keyword co-occurrence results intuitively reflect the research focus and population orientation of this field: “pressure injury” as the core keyword runs through the whole research process, while “elderly patients,” “risk factors,” and “advanced dementia” with high centrality indicate that the research objects are mainly concentrated in elderly and vulnerable groups with high risk of malnutrition, which is consistent with the clinical characteristics of high incidence of PI in these populations. The co-occurrence relationship between “enteral nutrition” and other core keywords further suggests that enteral nutrition intervention for high-risk groups has always been the core research direction in this field.

Keyword clustering results systematically reveal the core research themes and knowledge structure of PI nutritional support research. The largest cluster focuses on the basic prevention and risk assessment of PI, which is the foundation of this field; the micronutrient and wound healing cluster reflects the long-term attention to the nutritional mechanism of wound repair; the critical care and enteral nutrition cluster reflects the expansion of research to critically ill patients in recent years; the palliative care cluster reflects the transformation of research goals from simple wound healing to improving the quality of life of end-stage patients. The contour values of each cluster are all greater than 0.76, indicating that the clustering results are reliable and the research themes are clear and independent.

#### 4.3.2 Keyword clustering and core themes

Based on the LLR algorithm, 10 keyword clusters with contour values >0.76 were generated (Figure 9), indicating a clear, stable research structure (17) covering the entire PI nutritional support process: ① Basic prevention and control of Pressure Injuries (0.962, 29 keywords): The largest cluster, focusing on risk assessment, hospital-acquired PI prevention and care bundles, with core tools (Braden Scale) recommended as first-line prevention measures (1, 23); ② Micronutrients and wound healing (0.986, 2003): Extending to acute care, focusing on ICU patient nutrition and prone ventilation management, representing a major research cluster in critical care nutrition publications (4, 24); ③ Critical care and enteral nutrition (0.756, 2016): Extending to acute care, focusing on ICU patient nutrition and prone ventilation management, representing a major research cluster in critical care nutrition publications (1, 4, 22, 25); ④ Palliative care and advanced-stage intervention (0.957, 2018): Covering medical device-related PI prevention and jejunal tube feeding, mirroring the rising publication focus on end-stage care and quality of life, driven by global aging [Between 2015 and 2050, the proportion of the world's population over 60 years will nearly double from 12 to 22% (26)] and the growing demand for palliative care to enhance comfort and quality of life in end-stage patients [Each year, an estimated 56.8 million people, including 25.7 million in the last year of life, are in need of palliative care (27)]—reflecting a shift from wound healing to end-stage patient quality of life (16). These four dominant clusters reflect the major knowledge modules and evolutionary trends derived from bibliometric analysis.

It is important to acknowledge potential conceptual overlap among some keyword clusters (e.g., “nutritional support” and “critical care”). These clusters, derived algorithmically (LLR), may not be mutually exclusive in practice but reflect the integrated nature of this research field. Readers should interpret cluster distinctness with this limitation in mind.

#### 4.3.3 Phased research evolution (1994–2026)

PI nutritional support research showed distinct phased characteristics (Figure 9): 1994–2010 focused on basic nutritional assessment and risk identification, laying the field foundation; 2011–2015 centered on nursing standardization and evidence translation, with core research on clinical guideline formulation (16); 2016–2026 expanded toward precision nutrition in critical care (4, 28), palliative care and micronutrient metabolism monitoring, reflecting an individualization and precision trend driven by precision medicine (4).

The temporal evolution of research hotspots shows that the development of PI nutritional support research has gone through three clear stages. The early stage focused on the correlation between malnutrition and PI, laying a theoretical foundation for subsequent research; the middle stage focused on the standardization of nursing practice and the formulation of clinical guidelines, promoting the transformation of research results into clinical practice; the recent stage focused on precision nutrition and individualized intervention, reflecting the progress of research toward refinement and personalization. This phased development trend is closely related to the progress of clinical nutrition, wound care and precision medicine.

### 4.4 Research frontiers (2021–2026) *via* citation burst analysis

**Figure 13:**
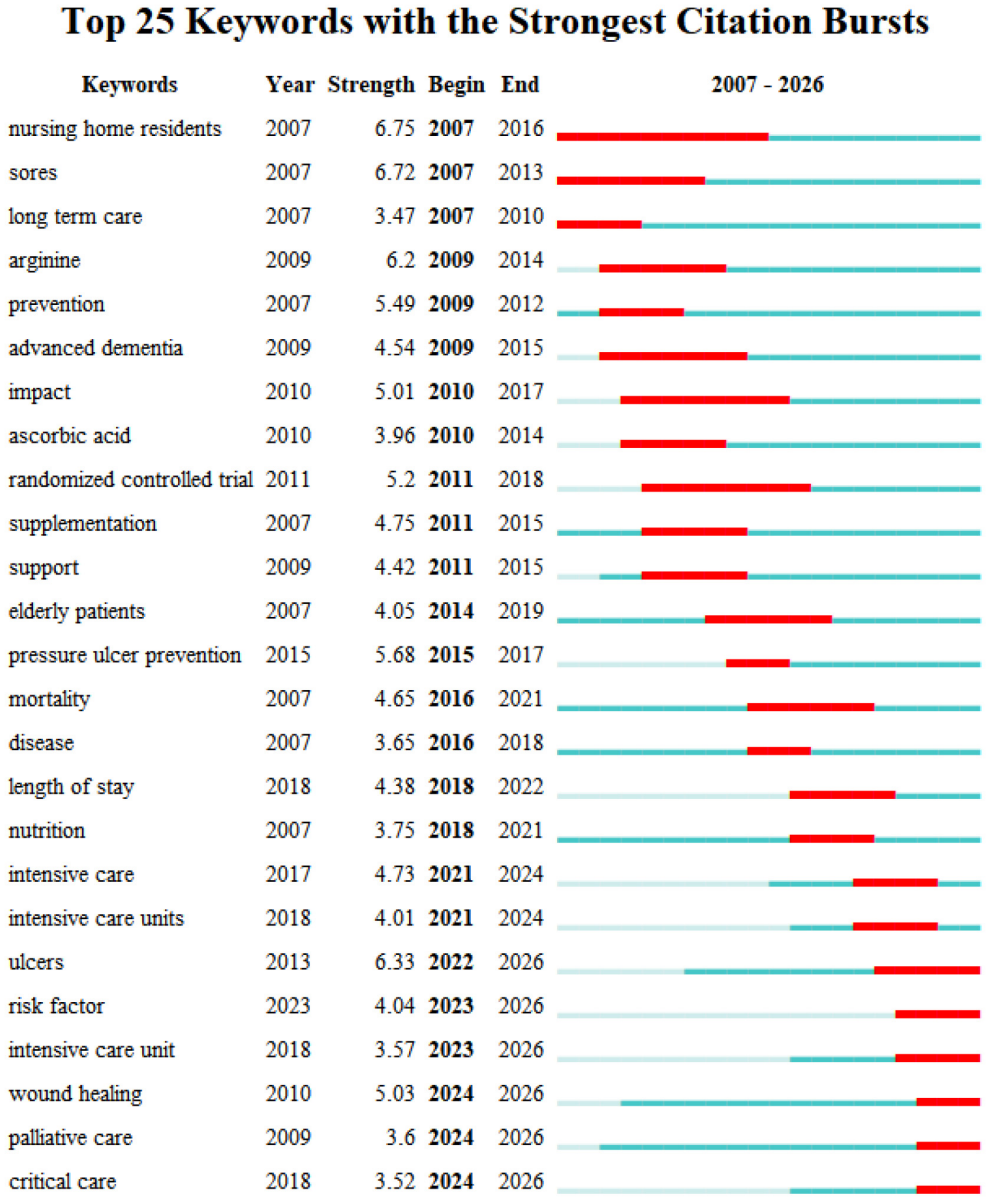
Top 25 keywords with the strongest citation bursts in the field of PI nutrition support.

Citation burst analysis identified 25 high-frequency burst keywords (Figure 13), While caution is needed in interpreting bursts as definitive “frontiers,” they highlight topics of recent and intensified interest. These include Intensive care (2021–2026) as the core long-duration burst keyword, marking ICU-acquired PI nutrition as a persistent hotspot driven by high ICU PI incidence and nutrition-clinical outcome correlations^6^ (22); Ulcers and risk factor (2022–2026) as the latest ongoing bursts, reflecting a return to PI risk stratification and pathophysiology research, aligned with the 2025 NPUAP guideline (1); Wound healing and palliative care (2024–2026) as the newest emerging bursts, linked to micronutrient metabolism research (24) and filling the end-stage patient nutrition gap (16). Burst keywords including micronutrients and protein-based nutrients confirm nutrient precision intervention as a persistent frontier (29–31), with current research concentrated on acute/critically ill populations and a clear disease specificity → standardized assessment → precise intervention trend.

Citation burst analysis can effectively identify the dynamic changes and emerging frontiers of research hotspots in the field. The results show that intensive care-related research has become the most lasting research hotspot in recent years, reflecting the high attention paid to the nutritional management of ICU patients with PI; wound healing and palliative care have become the latest emerging frontiers, indicating that the research focus has expanded from acute treatment to end-stage care; micronutrient-related keywords have always been in a burst state, confirming that precise nutritional intervention has been the core direction of recent research. These emerging frontiers reflect the latest development trend and research focus of PI nutritional support research.

Compared with previous bibliometric studies in this field, the present study not only confirmed that elderly patients, critical care, and enteral nutrition remain core themes but also newly identified palliative care, wound healing, and precise micronutrient intervention as emerging frontiers between 2024 and 2026. These findings expand and update the evidence base derived from earlier reviews and guideline-based studies.

### 4.5 Theoretical and practical implications for future research

This study clarifies the knowledge structure and evolutionary trends of nutritional support research for PI from database inception to February 2026, forming a systematic “risk identification → nutritional assessment → precise intervention” framework that aligns with international guidelines and contemporary high-quality clinical evidence (1, 16). By synthesizing nearly 30 years of global research trends, this work complements and updates existing thematic analyses and provides a more robust theoretical foundation for future translational studies.

From a theoretical perspective, this study systematically sorts out the knowledge structure, research hotspots and evolution rules of PI nutritional support research, clarifies the development context and disciplinary cross characteristics of this field, and provides a macroscopic perspective and theoretical reference for subsequent research.

From a research perspective, our bibliometric findings identify several promising avenues for future investigation. For instance, the observed trends suggest that future clinical studies could focus on validating the efficacy of personalized protocols and precision nutrition strategies. Consequently, any potential clinical application of these findings would be premature at this stage. Instead, our results can guide researchers in designing hypothesis-driven, interventional studies to test whether targeted nutritional support for high-risk populations (e.g., ICU, palliative care patients) translates into better clinical outcomes.

The trends we observed—such as the emphasis on early nutritional intervention and targeted micronutrient supplementation—are consistent with previously published clinical investigations (22, 32). However, bibliometric analysis itself does not provide evidence of clinical efficacy. Therefore, our findings should be interpreted as hypothesis-generating rather than practice-changing.

Furthermore, the increased research attention on palliative care and precision nutrition identified in this study aligns with calls for personalized nutrition care (33), highlighting a gap that future randomized controlled trials should address. Multidisciplinary collaboration remains a frequently cited theme in the literature, and our results underscore its continued emphasis in research discourse.

The innovation of this study lies in its comprehensive bibliometric visualization covering publication trends, international collaboration, keyword clustering, and citation bursts over nearly 3 decades. Different from traditional clinical research focusing on intervention effect, this study reveals the overall development status and future direction of the field through quantitative analysis of literature data, which provides a new research perspective for PI nutritional support research. By connecting macro-level research trends with existing clinical evidence (1, 16, 22, 32, 33), these results can help researchers prioritize questions for future high-quality studies.

## 5 Limitations

This study has several limitations. First, only English articles from the Web of Science Core Collection and PubMed were included, which may cause publication bias and ignore research from non-English-speaking countries. Second, bibliometric analysis relies on author-selected keywords, and their subjectivity may reduce the accuracy of identifying research hotspots and frontiers. Third, only the publication characteristics of literature were analyzed, without in-depth qualitative analysis of core literature content. Fourth, some RCTs in this field have methodological flaws (small sample sizes, inadequate randomization), which weakens the evidence strength and clinical translation of relevant conclusions. Fifth, sensitivity analysis across additional databases (e.g., Scopus) was not performed, and future studies could expand to multiple databases to further validate the robustness of the findings.

## 6 Future research directions

Based on the results up to February 2026 and existing limitations, future PI nutritional support research should focus on: conducting large-sample, multicenter RCTs to address methodological flaws (11); combining precision medicine and machine learning to develop personalized protocols for special populations (33); accelerating translation of high-level evidence into standardized clinical pathways; enhancing international and cross-disciplinary collaboration; and focusing on palliative care and health economic evaluation of nutritional interventions (19).

## 7 Conclusion

From database inception to February 2026, PI nutritional support research has maintained steady growth, becoming a key direction in geriatric care and chronic wound management, with a clear structural framework covering basic prevention, micronutrient intervention, critical care nutrition and palliative care. The latest research frontiers focus on ICU-acquired PI support, micronutrient precision intervention, wound healing and end-stage palliative care, presenting an individualization, precision and multidisciplinary collaboration trend. The “risk identification → nutritional assessment → precise intervention” theoretical system formed in this study is consistent with high-level evidence and may inform clinical practice, though the field still faces insufficient high-quality RCT evidence, incomplete special population research and inadequate research translation. Future research should prioritize high-quality clinical studies, personalized strategy exploration and international cross-disciplinary collaboration, integrating evidence-based medicine into standardized clinical pathways to optimize PI nutritional risk assessment and intervention, continuously improving clinical effects and patient quality of life.

## Data Availability

The original contributions presented in the study are included in the article/supplementary material, further inquiries can be directed to the corresponding author.
