# Supplementary material for: Nutritional support for pressure injury: a bibliometric analysis of research trends
Source: Front Nutr. 2026 Jul 2;13:1839197. doi: 10.3389/fnut.2026.1839197 (PMC13372707; doi:10.3389/fnut.2026.1839197)
Supplement: Supplementary file 1 [file Supplementary_file_1.docx]

**Supplementary File 1 Complete Executable Search Strings**

**Date of search**: February 28, 2026

**Database versions:**

Web of Science Core Collection (accessed via institutional subscription)

PubMed (https://pubmed.ncbi.nlm.nih.gov/)

**Web of Science Core Collection (WOSCC)**

TS=((“Nutritional intervention” OR “Dietary intervention” OR “Nutritional support” OR “Dietary support” OR “Nutritional therapy” OR “Dietary therapy” OR “Nutritional management” OR “Dietary management” OR “Nutritional supplementation” OR “Dietary supplementation” OR “Nutritional care” OR “Dietary care” OR “Medical nutrition therapy” OR “Therapeutic nutrition” OR “Enteral nutrition” OR “Parenteral nutrition” OR “Protein supplementation” OR “Vitamin supplementation” OR “Micronutrient intervention” OR “Oral nutritional supplements” OR “Tube feeding”) AND (“Pressure ulcer*” OR “Bed sore*” OR “Decubitus ulcer*” OR “Pressure sore*” OR “Decubitus sore*” OR “Pressure injur*”))

**PubMed**

(“nutritional intervention”[Title/Abstract] OR “dietary intervention”[Title/Abstract] OR “nutritional support”[Title/Abstract] OR “dietary support”[Title/Abstract] OR “nutritional therapy”[Title/Abstract] OR “dietary therapy”[Title/Abstract] OR “nutritional management”[Title/Abstract] OR “dietary management”[Title/Abstract] OR “nutritional supplementation”[Title/Abstract] OR “dietary supplementation”[Title/Abstract] OR “nutritional care”[Title/Abstract] OR “dietary care”[Title/Abstract] OR “medical nutrition therapy”[Title/Abstract] OR “therapeutic nutrition”[Title/Abstract] OR “enteral nutrition”[Title/Abstract] OR “parenteral nutrition”[Title/Abstract] OR “protein supplementation”[Title/Abstract] OR “vitamin supplementation”[Title/Abstract] OR “micronutrient intervention”[Title/Abstract] OR “oral nutritional supplements”[Title/Abstract] OR “tube feeding”[Title/Abstract]) AND (“pressure ulcer”[MeSH Terms] OR “pressure ulcer*”[Title/Abstract] OR “bed sore*”[Title/Abstract] OR “decubitus ulcer*”[Title/Abstract] OR “pressure sore*”[Title/Abstract] OR “decubitus sore*”[Title/Abstract] OR “pressure injur*”[Title/Abstract])
